# Clinical significance of No. 10 and 11 lymph nodes posterior to the splenic vessel in D2 radical total gastrectomy: An observational study

**DOI:** 10.1097/MD.0000000000004581

**Published:** 2016-08-12

**Authors:** Wei Wang, Wenjun Xiong, Zhiwei Liu, Lijie Luo, Yansheng Zheng, Ping Tan, Dechang Diao, Liaonan Zou, Jin Wan

**Affiliations:** Department of Gastrointestinal Surgery, Guangdong Provincial Hospital of Chinese Medicine, the Second Affiliated Hospital of Guangzhou University of Chinese Medicine, Guangzhou, China.

**Keywords:** D2 lymphadenectomy, gastric cancer, laparoscopy, splenic hilum lymph node dissection, total gastrectomy

## Abstract

D2 lymphadenectomy is widely performed for advanced proximal gastric cancer, but complete dissection of No. 10 and 11 lymph nodes (LNs) is technically challenging, especially for those posterior to the splenic vessel. This study aimed to investigate the clinical significance of removing No. 10 and 11 LNs posterior to the splenic vessel in radical total gastrectomy. Between January 2013 and February 2015, 53 patients who underwent spleen-preserving D2 radical total gastrectomy were enrolled. While dissecting No. 10 and 11 LNs, we divided them into 2 parts, namely LNs anterosuperior and posterior to the splenic vessel, and the pathological data were reviewed. Sixteen patients underwent laparoscopy and 37 underwent laparotomy. No mortality was recorded. According to the pathological results, the TNM stages of the tumor were IIA in 11 patients (20.8%), IIB in 5 (9.4%), IIIA in 7 (13.2%), IIIB in 10 (18.9%), and IIIC in 20 (18.9%). The mean number of LNs retrieved was 30.3 ± 12.3. The sum of No. 10 and 11 LNs posterior to the splenic vessel was 59 and the mean number was 1.11 ± 1.47. One LN with metastasis was found in the special 59 regional LNs, and the metastasis rate was 1.9% (1/53). Concerning the low metastasis rate (1.9%) and difficult complete dissection of No. 10 and 11 LNs posterior to the splenic vessel, our initial analysis suggests that the rate of No. 10 and 11 LNs posterior to the splenic vessel metastasis was 1.9%, but further studies are needed to reveal its clinical significance in D2 radical total gastrectomy for advanced proximal gastric cancer.

## 1 Introduction

Gastric cancer is one of the most common digestive tract malignancies. ^[1]^ Based on current clinical evidence, radical resection surgery, including adequate gastrectomy and lymphadenectomy, is the only potentially curative measure for gastric cancer. The main transfer route of gastric cancer cell is lymph nodes (LN) metastasis, which is an important poor prognosis factor. Unfortunately, the lymphatic system of the stomach is special and complex, so standard lymphadenectommy is complicated and technique challenging.

In recent years, the incidence of proximal gastric cancer was gradually increasing. ^[2]^ For advanced tumors in the upper-third of the stomach, complete dissection of LNs along the splenic artery (No. 11) and the splenic hilum (No. 10) was indicated in Japanese Gastric cancer treatment guidelines. ^[3]^ Before 21st century, total gastrectomy (TG) combined with splenectomy and distal pancreatectomy was recommended as the classic procedure for completely removing these special LNs.[ ^4^ ^5^] However, a number of latest studies have reported that these prophylactic extended resections may result in a relatively high morbidity and mortality rates and the uncertain benefit on patient survival. [^6^ ^7^ ^8^] Undoubtedly, complete removal of the No. 10 and 11 LNs is technically challenging, irrespective of open or laparoscopic surgery, due to the tortuous splenic vessels and the high possibility of injury to the parenchyma of the spleen, especially for those posterior LNs along the splenic vessel and splenic hilum. In our previous study, we had explored the safety and feasibility of laparoscopic spleen-preserving splenic hulim LN dissection. ^[9]^

The LN classification criterion of gastric cancer had been sustained changed for guiding postoperative therapy and more accurately for estimating the prognosis. In the present study, we performed standard radical TG with pancreas and spleen-preserving No. 10 and 11 LN dissection to investigate the technical characteristics of posterior No. 10 and 11 LNs and analyze this special LNmetastasis.

## 2 Materials and methods

### 2.1 Patient selection

Between January 2013 and February 2015, 53 patients with upper third or middle third gastric cancer underwent TG with spleen-preserving D2 LN dissection at the Department of Gastrointestinal Surgery, the Second Affiliated Hospital of Guangzhou University of Chinese Medicine. The indications for this procedure were as follows: the tumor was located in the upper third or middle third of the stomach, and T stage was T2 to T4a based on preoperative examination according to Japanese classification of gastric carcinoma (third English edition). ^[10]^ Patients with a distant metastasis, or apparent nodal metastasis in the splenic hilum or along the splenic artery based on preoperative examination or operative exploration were excluded. The endoscopic ultrasound and abdominal high-resolution multidirectional computed tomography was performed to estimate the preoperative tumor stage for all patients. All patients were notified with details about the operative procedure and potential risks before operation, and the informed consent was obtained. This study protocol was approved by the ethics committee of Guangdong Provincial Hospital of Chinese Medicine.

### 2.2 Special definition

**Figure 1:**
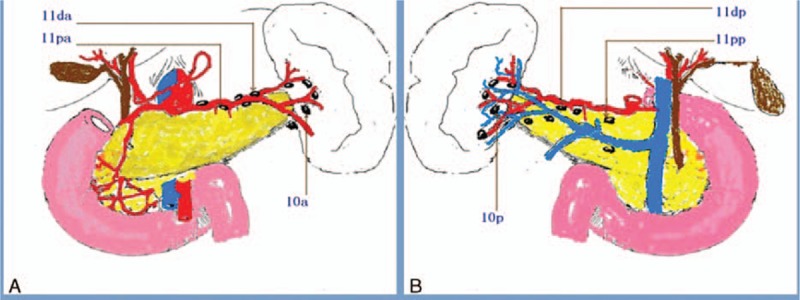
Abridged general view. A, Anterior view; B, posterior view. a = Artery, v = vein.

During dissection of No. 10 and 11 LNs, we divided them into 2 parts, respectively, namely LNs anterosuperior and posterior to the splenic artery or the splenic hilum, and the exact definition of these LNs according to Japanese classification of gastric carcinoma ^[10]^ was as follows (Fig. 1):

1. 10a: anterosuperior LNs along the splenic artery distal to the pancreatic tail, and those on the roots of the short gastric arteries and those along the left gastroepiploic artery proximal to its first gastric branch
2. 10p: posterior LNs along the splenic artery distal to the pancreatic tail
3. 11pa: anterosuperior LNs along the proximal splenic artery from its origin to halfway between its origin and the pancreatic tail end
4. 11da: anterosuperior LNs along the distal artery from halfway between its origin and the pancreatic tail end to the end of the pancreatic tail
5. 11pp: posterior LNs along the proximal splenic artery from its origin to halfway between its origin and the pancreatic tail end
6. 11dp: posterior LNs along the distal artery from halfway between its origin and the pancreatic tail end to the end of the pancreatic tail.

### 2.3 Laparoscopic surgery procedures

The procedure was carried out under general anesthesia with endotracheal intubation. The patient lay on the table in the supine position, with legs apart and 20° head-up tilt. CO_2_ pneumoperitoneum is induced after insertion of the first 10 mm trocar at the level of the umbilicus. Exploration of the abdominopelvic cavity was conducted to exclude distant metastasis. Four other working ports are inserted through the abdominal wall. The order of LN dissection was No. 4–6–7, 8a, 9, 12a, 5–10, 11-1, 2, 3, and the operative details were described in our previous article. ^[9]^

**Figure 2:**
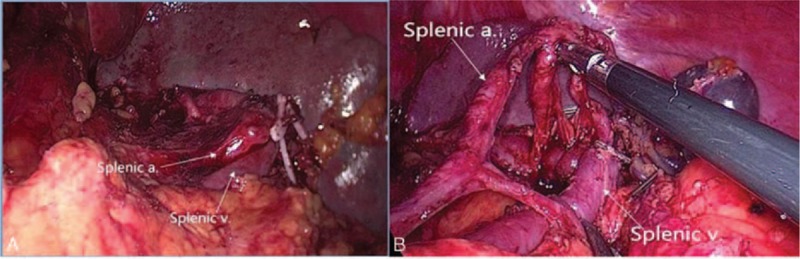
A, Anterosuperior LNs of No. 11 and No. 10 dissection in laparoscopic surgery. B, Posterior LNs of No. 11 and No. 10 dissection in laparoscopic surgery. a = Artery, LN = lymph node, v = vein.

Finally, the anterosuperior lymphatic fatty tissue of No. 11d and No.10 were dissected thoroughly (Fig. 2A), and the LNs No. 11 and No. 10 were completely removed, and all vessels in the splenic hilum area were saved (Fig. 2B).

A Roux-en-Y esophagojejunostomy were carried out intracorporeally using a circular stapler. An end-to-side jejunojejunostomy was performed.

### 2.4 Open surgery procedures

**Figure 3:**
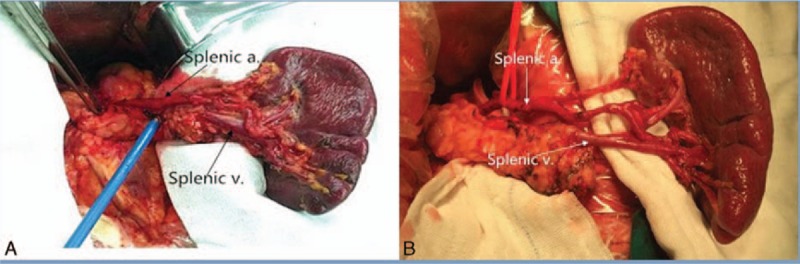
A, Anterosuperior LNs of No. 11 and No. 10 dissection in open surgery. B, Posterior LNs of No. 11 and No. 10 dissection in open surgery. a = Artery, LN = lymph node, v = vein.

No. 10 and 11 LN dissection after the splenocolic ligament and phrenicosplenic ligament was divided. The spleen and pancreatic tail were mobilized and put out of the abdominal cavity. An assistant hold the spleen. Thereafter the left gastroepiploic vessels and short gastric vessels were transected at their roots. The splenic artery was skeletonized from the distal to the proximal end. The anterosuperior lymphatic fatty tissue of No. 11d and No. 10 were dissected thoroughly (Fig. 3A). The spleen and the pancreatic tail were overturned to expose the posterior lymphatic fatty tissue of spleen and pancreatic tail skeletonizing. Then, those lymphatic fatty tissues were dissected from the distal to the proximal end. The LNs of No. 10p, 11pp, and 11dp were dissected (Fig. 3B). The details of the open surgery procedure was given in our previous study. ^[11]^

### 2.5 Postoperative therapy

Adjuvant chemotherapy was delivered for all patients. The regimen was XELOX: eight 3-week cycles of oral capecitabine (1000 mg/m^2^ twice daily on days 1 to 14 of each cycle) plus intravenous oxaliplatin (130 mg/m^2^ on day 1 of each cycle) for 6 months. ^[12]^

### 2.6 Data analysis

Data are expressed as either mean ± standard deviation or median with range. All analyses were performed with SPSS version 17.0 (Chicago, IL).

## 3 Results

The clinicopathological characteristics of the patients are shown in Table 1. There were 33 male and 20 female patients, with a mean age of 58.9 ± 11.1 years. The mean body mass index was 21.4 ± 2.6 kg/m^2^. The mean tumor size was 4.0 ± 1.9 cm, with 43 tumors located in the upper-third of the stomach and 10 in the middle-third.

**Table 1:**
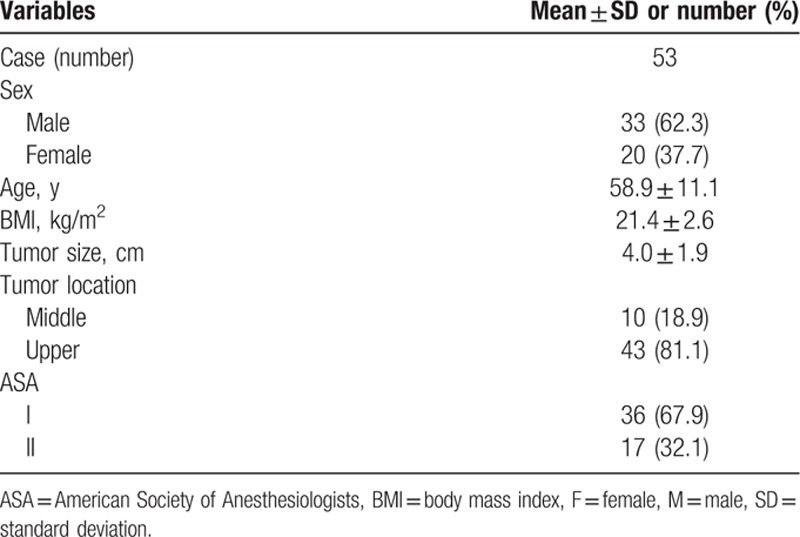
Clinicopathological characteristics of patients.

Surgical outcomes and postoperative course are summarized in Tables 2 and 3. Radical TG with pancreas and spleen-preserving splenic hilum LN dissection was successfully performed in all 53 patients. Sixteen patients underwent laparoscopy and 37 underwent laparotomy. The intraoperative morbidity was 9.4% (5/53): 4 intraoperative bleeding during the skeletonization of the splenic artery and splenic vein, and 1 splenic parenchyma injury. The postoperative morbidity rate was 11.3% (6/53): 1 anastomotic hemorrhage, 1 pancreatic fistula and 4 pulmonary infections. The mean number of retrieved LNs was 30.3 ± 12.3 and the mean number of retrieved 10a LNs was 2.72 ± 1.43, and 5 patients had 10a LN metastasis (9.4%). The mean number of retrieved LNs of No. 11pp, 11dp, and 10p was 1.11 ± 1.47, and only 1 patient had LN metastasis (1.9%), who had 10a LNs metastasis too. In accordance with the seventh edition American Joint Committee on Cancer (AJCC) cancer staging manual, the TNM stages of those patients were distributed as follows: 11 stage IIA, 5 stage IIB, 7 stage IIIA, 10 stage IIIB, and 20 stage IIIC.

**Table 2:**
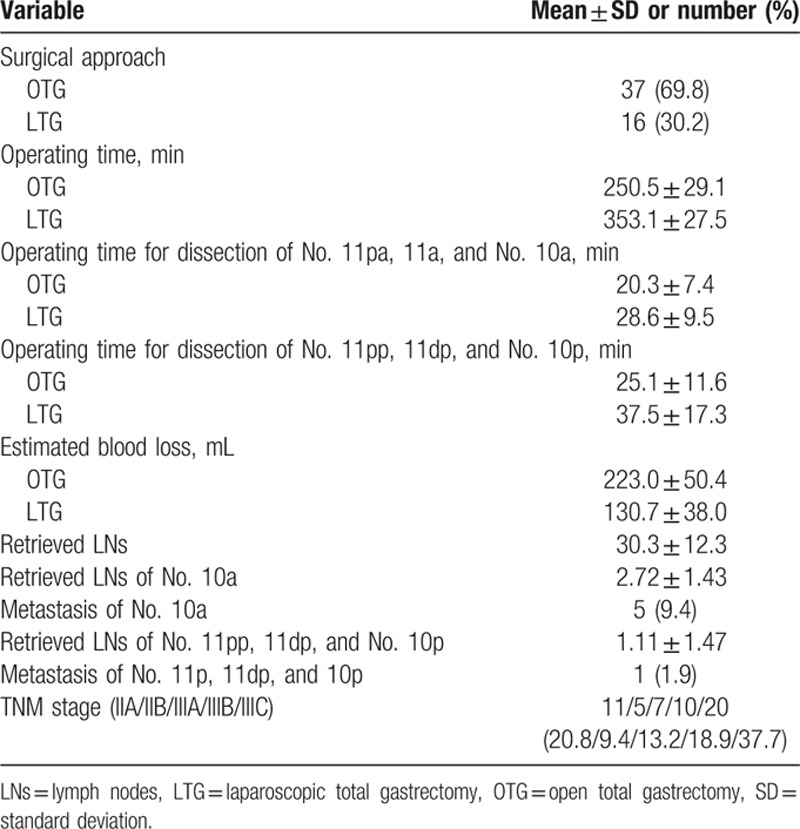
Intraoperative and postoperative outcomes.

**Table 3:**
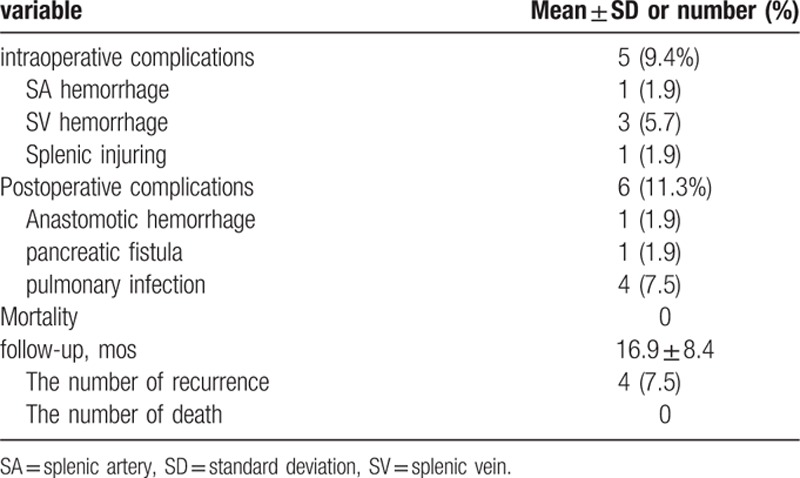
Overall complications and follow-up.

After a median follow-up of 16.9 months (range 3–29 months), 4 patients appeared with peritoneal metastasis. The only 1 patient with No. 11pp, 11dp, and10p LN metastasis had peritoneal metastasis in 11 months after operation and was receiving basic supportive care.

## 4 Discussion

Lymph node metastasis is an important prognosis factor of gastric cancer. Radical gastrectomy is the only curative treatment option for patients with gastric cancer. An adequate resection margin and systematic lymphadenectomy are essential for radical gastrectomy. However, the lymphatic system of the stomach is complicated and complete removal of LNs in radical gastrectomy is difficult. In TG for proximal gastric cancer, No. 10 and 11 LN dissection was recommended, which was most technically challenging in radical gastrectomy, especially for those LNs posterior to the splenic vessel. In present study, we had investigated the technique characteristics of posterior No. 10 and 11 LNs, and the special LNs metastasis rate was 1.9%.

The changes and controversies of LN classification criterion of gastric cancer persisted for guiding postoperative therapy and more accurately for estimating the prognosis. To investigate the rule of LN metastasis of gastric carcinoma, a large number of difficult and endless studies were undertaken. In 1985, the No. 8 LNs were divided into 2 subgroups: 8a (anterosuperior LNs along the common hepatic artery) and 8p (posterior LNs along the common hepatic artery), because for the No. 8a LNs, a higher metastatic rate was observed in the clinical study when compared with the No. 8p LNs. From the first “Gastric cancer treatment guidelines in Japan” in 1962, the development of “guidelines” has already passed half a century until now. However, the investigation was still going on. In the 14th “Gastric cancer treatment guidelines in Japan,” the No. 3 LNs were divided into 2 subgroups: 3a (lesser-curvature LNs along the branches of the left gastric artery) and 3b (lesser-curvature LNs along the second branch and distal part of the right gastric artery). ^[10]^ The purpose was to observe the relationship between the 2 subgroups and the location of gastric cancer, especially for the early tumor.

The dissection of No. 10 and 11 LNs in gastric cancer surgery is indispensable for treating advanced gastric cancers located in the proximal third of the stomach. Spleen and pancreas-preserving splenic hilum LN dissection was the best option for a patient with advanced proximal gastric cancer without apparent nodal metastasis in the splenic hilum or spleen or pancreatic involvement. However, because of the tortuous splenic vessels and the high possibility of injury to the parenchyma of the spleen, spleen-preserving D2 LN dissection is not a simple technique, especially for the dissection of LNs posterior to the splenic vessel. During dissecting No. 10 and 11 LNs, we divided them into 2 parts, namely LNs anterosuperior and posterior to the splenic vessel.

In our initial study, the dissection of LNs anterosuperior and posterior to the splenic vessel was safe and feasible. The anterosuperior lymphatic fatty tissues of No. 11 and No. 10 were completely removed. The dissection of lymphatic fatty tissue posterior to the splenic vessel was more difficult and more intraoperative complication than those anterosuperior LNs. The metastasis rate of No. 10a was similar with the previous studies.[ ^13^ ^14^] The sum of retrieved No. 11pp, 11dp, and 10p LNs was 59; only 1 LN was metastasized. At present, there were no researches about the clinical significance of the No. 11pp, 11dp, and 10p LN dissection.

Complete dissection of No. 10 and 11 LNs is technically challenging, especially for LNs posterior to the splenic vessel, namely No. 11pp, 11dp, and 10p. Because of the deep location of spleen, narrow space, and wide variations in the distribution of the splenic vessels, the spleen-preserving D2 lymphadenectomy was difficult for both laparoscopic and open approach. In our study, 37 patients underwent laparotomy. A satisfactory result was achieved with the curettage and aspiration in spleen-preserving splenic hilar LN dissection. ^[11]^ There were some studies showing that a better short-term result of spleen-preserving D2 lymphadenectomy was recorded in the laparoscopy. [^15^ ^16^ ^17^] In our study, the safety of the 16 patients who underwent laparoscopy was similar to open surgery. During the laparoscopic operation procedure, we used the left approach, ^[18]^ from branches to the root of the splenic artery anterosuperior LNs of No. 11 and No. 10 dissection. The gross was then removed to offer a better space for the No. 11pp, 11dp, and 10p LN dissection. During the procedure, the splenic vessel was skeletonized. Due to the thin vascular wall, wide variations of the splenic vein, and narrow retropancreatic space, it was easy to injury the splenic vein and splenic parenchyma. In our study, 3 patients experienced intraoperative bleeding and 1 patient experienced spleen injury, but were handled successfully without intraoperative death. With the help of the suction/irrigation tubes and keeping ultrasonic scalpel's nonfunctional face close to the surface of the tissue were efficient measures to prevent injury. The postoperative morbidity in our study was 12%, which was similar to postoperative morbidity of spleen-preserving splenic hilum dissection reported (range 5.1%–21.6%).[ ^13^ ^14^ ^16^]

Lymph node metastasis was the main transfer route of gastric cancer cell. To improve the effect of radical gastrectomy, the definition of systematic lymphadenectomy was first proposed by the Japanese. The Japanese Gastric Cancer Association had classified the perigastric regional LNs to investigate the rule of LN metastasis of gastric carcinoma in 1960. Until now, the 14th “guidelines” was published in 2010. Defining the LNs of the stomach reasonably was one of the major content of each revision. In the 13th “guidelines,” the No. 11 LN was divided into 2 subgroups: No. 11p and 11d. The 13th “guidelines” recommend that only the No. 11p LN dissection was necessary in the distal gastrectomy with D2 lymphadenectomy, because when the tumor was located in the distal stomach, the metastatic rate of No. 11d was much lower than those in No. 11p and not significant for dissection. On the contrary, the No. 12 LNs were divided into 3 groups: No. 12a, 12b, and 12p. The 12b and 12p LNs were defined as the N3 group. In the 13th “guidelines,” if the LN metastasis was detected in the N3, D3 surgery was needed and the prognosis was poor. Sasada et al ^[14]^ reported that No. 10 LN metastasis was the significant factor affecting prognosis. The metastasis rate of the splenic hilar LNs has been reported to range between 9% and 20.9% in proximal gastric cancer,[ ^4^ ^6^ ^7^] and the prognosis of the patient with No. 10 LN metastasis was poor. Some reports had showed that the 5-year survival rate of those patients with splenic hilar LN metastasis ranged from 11.04% to 22.2%, and 5-year survival rate of those patients without splenic hilar LN metastasis ranged from 49% to 51.57%.[ ^13^ ^19^] In our study, the metastasis rate of the No. 10a LNs was 9.4% and the metastasis rate of the No.10p, 11pp, and 11dp was 1.9%. The prognosis of the patient with No. 10p, 11pp, and 11dp LN metastasis was poor. Based on our initial results, some questions were raised: one is that whether it is necessary to dissect the No. 10p, 11pp, and 11dp LNs, and the other one is that if No. 10p, 11pp, and 11dp LN metastasis was detected, whether the D3 lymphadenectomy was required. Future studies are needed to resolve these questions.

In conclusion, the dissection of LNs anterosuperior and posterior to the splenic artery was safe and feasible for both laparoscopic and open surgery. No. 10p, 11pp, and 11dp LN complete dissection is technically challenging. In our initial study, the metastasis rate of the No. 10p, 11pp, and 11dp was 1.9%. Future studies were needed to investigate the clinical significance of removing No. 10p, 11pp, and 11dp LNs in radical TG.
